# Studies on Sensing Properties and Mechanism of CuO Nanoparticles to H_2_S Gas

**DOI:** 10.3390/nano10040774

**Published:** 2020-04-17

**Authors:** Fang Peng, Yan Sun, Yue Lu, Weiwei Yu, Meiying Ge, Jichao Shi, Rui Cong, Jiaming Hao, Ning Dai

**Affiliations:** 1 State Key Laboratory of Infrared Physics, Shanghai Institute of Technical Physics, Chinese Academy of Sciences, Shanghai 200083, China; zpfzyyx@163.com (F.P.); Lyue8730@163.com (Y.L.); taylorfish@163.com (W.Y.); congrui@mail.sitp.ac.cn (R.C.); jiaming.hao@mail.sitp.ac.cn (J.H.); 2 School of Electronic Electrical and Communication Engineering, University of Chinese Academy of Sciences, Beijing 100049, China; 3 School of Materials Science and Engineering, University of Shanghai for Science and Technology, Shanghai 200093, China; 4 National Engineering Research Center for Nanotechnology, No. 28 East Jiang Chuan Road, Shanghai 200241, China; meiyingge@163.com; 5 School of Materials Science and Engineering, Shanghai Institute of Technology, Shanghai 200235, China; jcshi@sit.edu.cn; 6 Hangzhou Institute for Advanced Study, University of Chinese Academy of Sciences, Hangzhou 310024, China

**Keywords:** CuO, H_2_S, mechanism, oxidation, CuS formation

## Abstract

In this work, the high crystalline copper oxide (CuO) nanoparticles were fabricated by a hydrothermal method, and their structural properties were characterized by transmission electron microscopy (TEM), X-ray diffraction (XRD), and X-ray photoelectron spectroscopy (XPS). The sensing results show that CuO nanoparticles exhibit enhanced sensitivity and good selectivity for hydrogen sulfide (H_2_S) gas at a low temperature. There are two working mechanisms involved in the H_2_S sensing based on CuO nanoparticle sensors. They are the H_2_S oxidation mechanism and the copper sulphide (CuS) formation mechanism, respectively. The two sensing mechanisms collectively enhance the sensor’s response in the H_2_S sensing process. The Cu–S bonding is stable and cannot break spontaneously at a low temperature. Therefore, the CuS formation inhibits the sensor’s recovery process. Such inhibition gradually enhances as the gas concentration increases from 0.2 ppm to 5 ppm, and it becomes weaker as the operating temperature rises from 40 °C to 250 °C. The XPS results confirmed the CuS formation phenomenon, and the micro Raman spectra demonstrated that the formation of CuS bonding and its decomposition can be effectively triggered by a thermal effect. Gas-sensing mechanism analysis supplied abundant cognition for the H_2_S sensing phenomena based on CuO materials.

## 1. Introduction

Hydrogen sulfide (H_2_S) gas is a highly poisonous and flammable gas with a rotten egg smell, which is widely prevalent in oil, coal mines, sewage plants, and natural gas industries. It can corrupt and irritate the eyes, skin, and respiratory system at a very low concentration of 10 ppm, and even causes paralysis and death when its concentration exceeds 120 ppm [1,2]. In the past several decades, stannic oxide (SnO_2_) [3], molybdenum oxide (MoO_3_) [4], zinc oxide (ZnO) [5], α-ferric oxide (α-Fe_2_O_3_) [6], tungsten oxide (WO_3_) [7], etc. typical n-type oxide semiconductors have been investigated for H_2_S gas detection. Recently, increasing interest has been taken in gas sensors based on p-type semiconductors, such as CuO, for its chemical stability, electrochemical activity, and high electron communication features [8]. CuO colloidal particles have been prepared by Duan and exhibit outstanding room-temperature sensing properties as low as 100 ppb H_2_S gas, through a complicated preparation process [9]. Kim et al. worked on Pd-functionalized CuO for H_2_S gas sensing and the sensor showed an enhanced response with a rapid recovery despite not working at a low temperature [10]. In this paper, small-sized CuO nanoparticles were prepared using a simple and inexpensive one-step hydrothermal method, which is suitable for the industrial production. In addition, the CuO nanoparticles sensors possess the response of 4.9 ± 0.43 ppm to 5 ppm H_2_S at 40 °C, a fast recovery time of 54 ± 7.1 s via heating pulse modulation, and an excellent sensing selectivity, which makes the sensor a promising candidate for practical applications.

Furthermore, CuO modified *n*-type oxide semiconductors, such as CuO/SnO_2_ and CuO/WO_3_, also show remarkable H_2_S sensing properties, especially in low temperature monitoring [11,12]. Understanding the working mechanism of CuO materials sense to H_2_S gas is conducive to clear functionalized CuO composites in H_2_S detection. Until now, the two sensing mechanisms of CuO material to H_2_S gas are recognized. The one H_2_S oxidation mechanism refers to the desorption process of oxygen ions O^δ−^ (e.g. O_2_^−^, O^−^, and O^2−^) that were pre-adsorbed on the surface of CuO material [13,14,15]. The related chemical equation is shown as Equation (1).

(1) $$H_{2}S(\mathrm{gas})+3O^{\delta -}(\mathrm{ads})\to H_{2}O(\mathrm{gas})+SO_{2}(\mathrm{gas})+3\delta e^{-}$$

The other CuS formation mechanism involves a chemical reaction accompanied by a change in the electrical potential [16,17,18]. The spontaneous chemical reaction is demonstrated as Equation (2).

(2) $$\mathrm{CuO}(s)+H_{2}S(g)\to \mathrm{CuS}(s)+H_{2}O(g)$$

The amazing performances are most attributed to the chemical transformation of highly resistive *p*-CuO into well conducting Cu_2_S or CuS, which leads to a drastic decrease in resistance [10,19].

However, it is well known that the two working mechanisms are involved in CuO material to H_2_S gas. However, there are still many problems in mechanism cognition to be solved. For example, many studies reported that the formation of CuS decreases the electrical resistance because CuS has the metallic character [12,18,20]. However, H_2_S molecules react with the pre-adsorbed oxygen species, which results in an increase in electrical resistance of p-type CuO [10]. Thereupon, it is still unclear whether the two sensing mechanisms are synergistic or antagonistic to the sensor response. On the other hand, CuS cannot be oxidized to CuO completely at a low temperature since the Cu–S bonding is too strong to break and, hence, prolongs the sensor’s recovery process [21,22]. Therefore, the urgent task is to regulate the recovery process hindered by CuS formation, and to understand whether such inhibition on the recovery process is affected by the operating temperatures and gas concentrations. Herein, this work makes up for above deficiencies in the mechanism analysis. In addition, the H_2_S sensing performances of CuO nanoparticles are also described.

## 2. Experimental Scheme

All the chemicals were of an analytical grade with no further purification, and all liquid reagents were purchased from Shanghai Chemical Industrial Co. Ltd. (Shanghai, China) and test gases were purchased from Shanghai Shenkai Gas Technology Co. Ltd (Shanghai, China). The gases included H_2_S/N_2_ (10%), CO/N_2_ (15%), Alcohol (CAS: 64-17-5, >99.7%), Acetone (CAS: 116-09-6, >99.8%), Methyl alcohol (CAS: 67-56-1, >99.5%), Ammonia water (CAS: 1336-21-6, ≥28%), Methane (CAS: 137829-79-9, 98%), and Isopropanol (CAS: 67-63-0, 99.9%).

### 2.1. Synthesis of CuO Nanoparticles

CuO nanoparticles were synthesized by a typical hydrothermal method. In total, 0.5 g of copper acetate monohydrate (Cu (CH_3_COO)_2_·H_2_O, A.R. Aladdin, Shanghai, China) was dissolved in 180 ml alcohol (C_2_H_5_OH, A.R. Aladdin, Shanghai, China) under stirring. Then, the solution was transferred to 100 mL Teflon-lined stainless autoclaves for hydrothermal reaction at 140 °C for 4 hours. Lastly, the CuO nanoparticles were separated by centrifugation and washed with deionized water and ethanol several times to remove the remnant in the mixture. Then, they were dried at 80 °C for 2 hours in the drying oven. 

### 2.2. Characterization of Structures and Morphologies 

The morphology of CuO nanoparticles were characterized by transmission electron microscopy (TEM) with selected-area electron diffraction (SAED) (JEOL2100F, JEOL, Tokyo, Japan). The crystal structure of the samples was investigated by Powder X-ray diffraction (XRD, D/max-2600PC, Rigaku Corporation, Tokyo, Japan) with Cu Kα radiation (λ = 1.5406 Å). To analyze the chemical bonds of samples, the X-ray photoelectron spectrometer (XPS, ESCALAB 250Xi, Thermo Fisher Scientific, Waltham, MA, USA) was carried out using Al KR X-ray as the excitation source, and Raman spectra (LabRam HR800 Ev, HORIBA Jobin Yvon, Paris, France) was employed at the excitation wavelength of 532 nm.

### 2.3. Preparation and Measurement of the Gas Sensor

**Figure 1:**
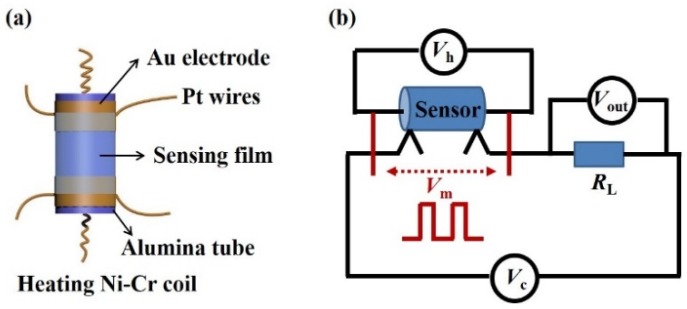
Schematic diagrams of (**a**) a sensor element and (**b**) testing circuit of the gas sensor.

The gas sensors are of a side-heating type with alumina tube structure, and the schematic diagram of the sensor element is shown in Figure 1a. A proper amount of CuO powder was uniformly coated on a ceramic tube as a sensing film layer. A Ni-Cr coil was set through the tube as a heater and the operating temperature was adjusted by the heating voltage. The gas sensing test was performed on the HW-30A system (Hanwei Electronics Co. Ltd., Zhengzhou, China).

The electronic circuit of the gas sensor is illustrated in Figure 1b. In this scenario, *V*_C_ is the test voltage with a certain value of 5 V, *V*_H_ is the heating voltage modulated on the Ni-Cr coil, *V*_m_ is the heating pulse voltage, *V*_out_ is the output voltage on *R*_L_, and *R*_L_ is a load resistor in series with the gas sensor. With the recorded *V*_out_ values of the load resistor, the equivalent resistances of the sensor can be calculated. The response of the sensor was determined by the value of R_g_/R_a_. The resistance of the sensor in testing gases (R_g_) was divided by that in air (R_a_). The response time and recovery time, respectively, refer to the times for the sensor output to reach 90% of its saturation after injection and release of the target gas, which are indicated as T_90_. All the measurements were carried out under the same condition of about 40% relative humidity.

## 3. Results and Discussions

### 3.1. Morphological and Structural Characteristics

**Figure 2:**
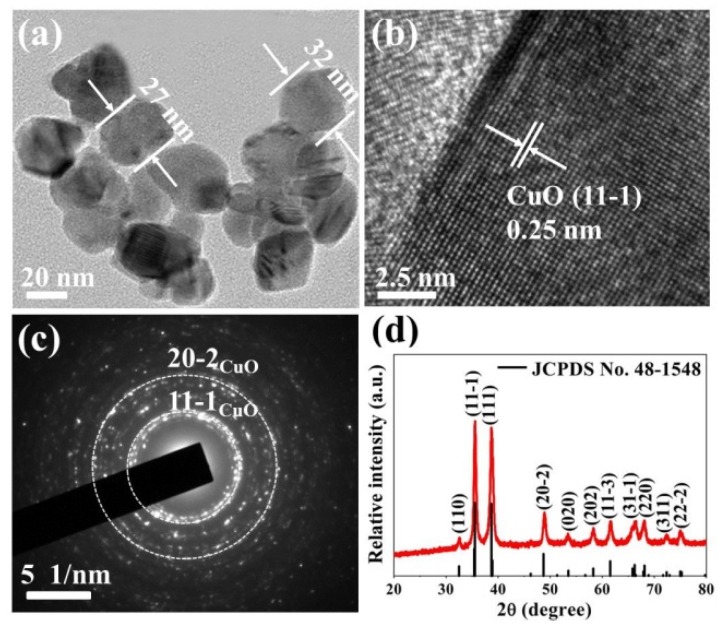
(**a**) Transmission electron microscopy (TEM) image, (**b**) High power transmission electron microscopy (HRTEM) image, (**c**) Selected-area electron diffraction (SAED) pattern, and (**d**) Powder X-ray diffraction (XRD) pattern of CuO nanoparticles.

The phase and structural features of CuO nanoparticles were analyzed by TEM and SAED. As shown in Figure 2a, the CuO nanoparticles are extremely small and their diameters are approximately in a range of 20–46 nm with stone-like morphology. Such a small particle size is compared to twice the Debye length of CuO reported to be ~12.7 nm [23]. According to the crystal size effect, this size will make the whole grain depleted and the resistance is controlled by the CuO nanostructure itself, which results in a high response [24]. Figure 2b illustrates the high-resolution TEM (HRTEM) image of CuO nanoparticles. The lattice fringes with inter-planar spacing of 0.25 nm match well with the (11-1) plane of simple monoclinic CuO. The SAED patterns in Figure 2c clearly show the ring pattern (circles marked in a white color) mainly arising from the (11-1) and (20-2) crystal indexes of CuO structures, which implies the crystalline nature of CuO nanoparticles. Figure 2d is the corresponding XRD pattern of CuO nanoparticles. The well-defined diffraction peaks match the monoclinic structure CuO diffraction data (JCPDS card no.48-1548). The main peaks at 35.6° and 38.7° are correspond to (11-1) and (111) crystal planes of CuO, which is coincident with the HRTEM results. The average particle size of CuO nanoparticles was calculated by using the Debye-Scherrer formula [25].

(3) $$D=\frac{k\lambda }{\beta \cos2\theta }$$

where *k* is an empirical constant equal to 0.94, λ is the wavelength of X-ray radiation (1.5406 Å), *β* is the full width at half maximum of the diffraction peak, and *θ* is the angular position of the peak. The particle size is calculated to be 28 ± 2 nm. Such a value matches well with the HRTEM results. The (11-1) main crystal plane in the XRD patterns is also present in the HRTEM images.

### 3.2. Gas Sensing Properties 

**Figure 3:**
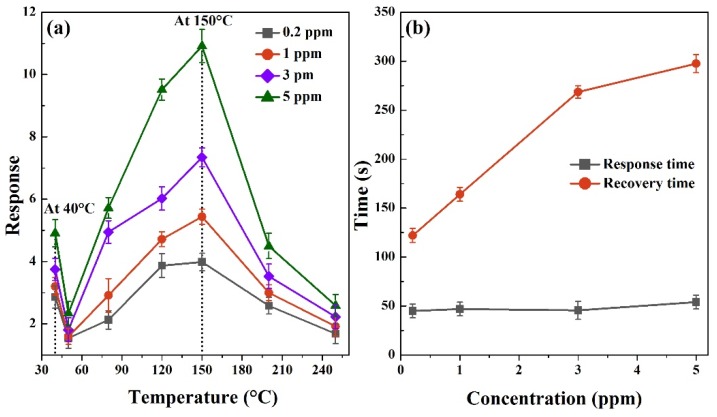
(**a**) The responses of CuO nanoparticles to 0.2, 1, 3, and 5 ppm H_2_S gas at different temperatures. (**b**) Response and recovery times of the sensor toward various H_2_S concentrations at 40 °C.

**Figure A1:**
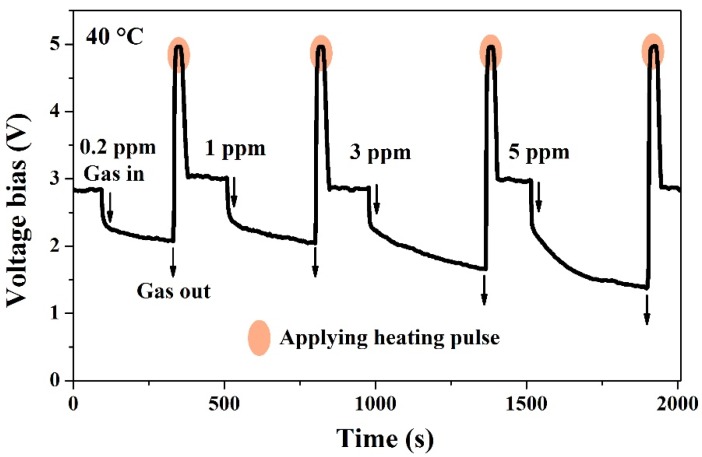
Real-time sensing curves of the CuO sensors to H_2_S gas (0.2, 1, 3, and 5 ppm, as labeled) at 40 °C.

Figure 3a displays the responses of CuO nanoparticles as a function of operating temperatures toward 0.2, 1, 3, and 5 ppm H_2_S gas. As seen, the responses exhibit the similar change trend to different H_2_S gas concentrations. The best working temperature is 150 °C at which the responses are 10.9 ± 0.53 and 4 ± 0.38 to 5 ppm and 0.2 ppm H_2_S gas, respectively. Even at 40 °C, the response reaches 4.9 ± 0.43 toward 5 ppm H_2_S gas and such a response is still suitable for monitoring the application. It has no doubts that the enhanced chemisorbed oxygen dominates the sensing process when the temperature is higher than 150 °C, since the Cu–S bonding decomposition can be effectively triggered by high temperature [21,22]. At a lower temperature of 40 °C, the sensing performance is mainly attributed to a CuS formation reaction. Since the oxygen adsorption and desorption process is suppressed at such a low temperature [13,14,15], its contribution to the device response is greatly diminished. The changing trend of response curves may result from the competition mode of the H_2_S oxidation mechanism and the CuS formation mechanism. The dynamic response processes of CuO nanoparticle sensors to various concentrations of H_2_S gas at 40 °C are recorded in Figure A1. Figure 3b shows the response and recovery times versus H_2_S concentrations at 40 °C. The response times increase form 122 ± 7.1 s to 297.5 ± 9.2 s since the H_2_S concentration increases from 0.2 ppm to 5 ppm. After applying a heating pulse, the recovery times decrease significantly within 54 ± 7.1 s, which indicates the rapid recovery feature at a low temperature. Our sensors have excellent sensing performance at 40 °C relative to the results reported previously, as shown in Table 1.

**Figure 4:**
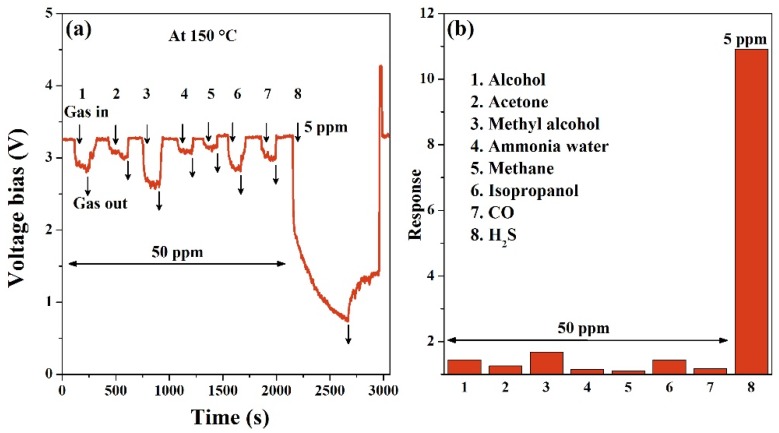
(**a**) Dynamic curves and (**b**) the responses of CuO nanoparticles to various gases at 150 °C.

The selectivity of the CuO nanoparticles sensor was measured to 5 ppm H_2_S gas and 50 ppm other gases, including alcohol, acetone, methyl alcohol, ammonia water, isopropanol, and carbon monoxide (CO). The tested results are displayed in Figure 4. The response dynamic curve in Figure 4a illustrates that *V*_out_ drops down to the response values upon 50 ppm various gases’ exposure (marked as 1–7), and it quickly returns to the baseline value after air purging at 150 °C. For the seven gases, the CuO sensor shows the rapid response of 30 ± 2 s and fast recovery of 50 ± 3 s. However, for 5 ppm H_2_S gas, the sensor’s response and recovery times are longer. The response histogram in Figure 4b demonstrates that the CuO sensor is more sensitive to a low concentration H_2_S gas than the other high concentration gases. The prominent selectivity plays a crucial role in the practical detection of hazardous H_2_S gas.

**Figure 5:**
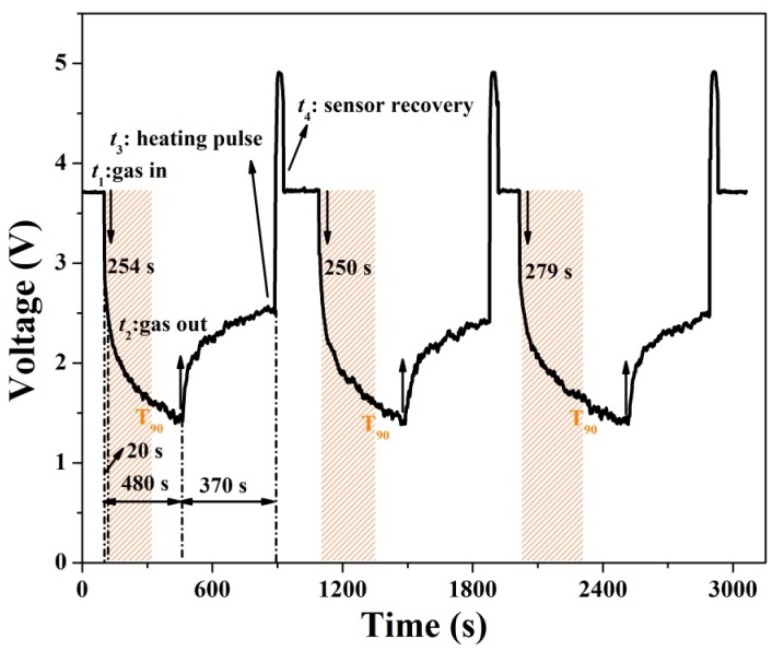
Dynamic response of CuO nanoparticles to 3 ppm H_2_S gas at 150 °C.

The dynamic measurement is the best way to examine the response process [27,28]. Figure 5 records the repeatability dynamic response toward 3 ppm H_2_S gas at 150 °C. When H_2_S gas is injected into the test chamber at *t*_1_, the reaction occurs spontaneously between H_2_S and the pre-adsorbed oxygen species, according to Equation (1). Therefore, *V*_out_ initially has a sharp decrease on H_2_S exposure within a short time (20 s). CuS formation occurs on the surface of the CuO nanoparticles, according to Equation (2), which causes the *V*_out_ continue to drift slowly to lower values (taking 480 s for the response value). The T_90_ position corresponding to each response stage has been marked in Figure 5. The average response time is 261 ± 3 s after the sensors are exposed to 3 ppm H_2_S gas. After switching off the H_2_S gas at *t*_2_, the oxygen species re-adsorbs on the CuO surface, which leads to the *V*_out_ rising up to a platform and recovering at about 25% within 370 s. Next, the *V*_out_ hardly keeps rising and returns to the baseline value due to CuS formation. Once a heating pulse (350 °C, 30 s) is applied on the sensor at *t*_3_, the *V*_out_ rapidly rises up to the baseline state within 40.3 ± 2 s at *t*_4_. The instant high temperature accelerates the complete conversion of CuS back to CuO [21,23], and the correlated reaction can be expressed by Equation (4).

(4) $$2\mathrm{CuS}+3O_{2}(g)\overset{\Delta }{\to }2\mathrm{CuO}(s)+2SO_{2}(g)$$

Such an operation has been successfully adopted in the previous works [10,23]. Thus, the sensor based on CuO nanoparticles presents stable and repeatable sensing properties with a heating voltage.

**Figure 6:**
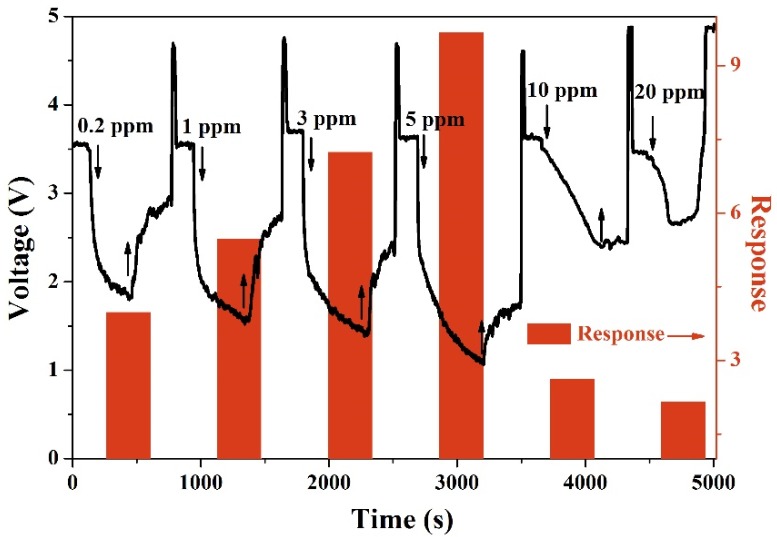
Real-time curves and the responses of CuO nanoparticles to varying amounts of H_2_S gas at 150 °C.

Furthermore, Figure 6 records the typical dynamic curves and responses on H_2_S concentrations ranging from 0.2 ppm to 20 ppm at 150 °C. Upon a lower concentration of H_2_S (0.2–5 ppm), the response is found to vary linearly with H_2_S concentration. Due to a rise in H_2_S concentration to 10 ppm, the reactions were increased and benefited from larger coverage, but the sensor’s response became weaker since the decline to *V*_out_ is smaller. This phenomenon violates the linear relationship between the response value and gas concentration. Further increasing H_2_S concentration to 20 ppm, the sensor’s response continues to decrease and the dynamic curve shows a unique feature. Initially, the *V*_out_ decreases slowly, which is followed by “a valley,” and increases sharply before removal of H_2_S gas. The device appears to be temporarily poisoned, and can only recover to the baseline state after applying multiple pulses. The performance may be attributed to the metallic CuS layer that forms on the CuO surface and the carrier flows directly from the CuS layer. Therefore, the sensor’s resistance decreases and, hence, the *V*_out_ increases. This phenomenon is consistent with the results observed by Ramgir [26]. Since the abnormal behavior occurs upon the sensor being exposed to a high concentration of H_2_S gas, we chose the sensor toward low concentrations of H_2_S gas (0.2–5 ppm) for further discussion.

### 3.3. Sensing Mechanisms

**Figure 7:**
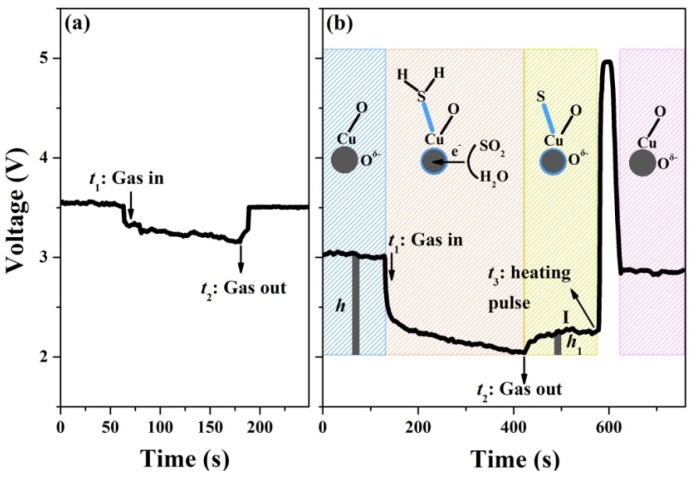
Dynamic curves of the CuO sensor toward (**a**) 300 ppm CO at 80 °C and (**b**) 1 ppm H_2_S at 40 °C.

To analyse the dependence of dynamic variation on the two sensing mechanisms, we chose to compare the real-time curves of CuO-based sensors to H_2_S gas and to CO gas, which is a reducing gas similar to H_2_S gas. Figure 7 presents the dynamic response in the presence and absence of 300 ppm CO and 1 ppm H_2_S gas. As seen in Figure 7a upon 80 °C, the *V*_out_ on *R*_L_ reduces to the response value after CO gas injection at *t*_1_. It spontaneously rises up to the baseline value within 30 s after the CO gas is released at *t*_2_. Based on the previous reports [29,30,31], the sensing performance of CuO material to CO gas is mainly induced by the oxidation mechanism based on depletion theory. The dynamic performance upon the CO exposure/release process displays that the oxygen adsorption process can be completed spontaneously, since the *V*_out_ returns to the baseline state rapidly after the CO release. As shown in Figure 7b, once the CuO nanoparticles sensor is exposed to H_2_S gas at *t*_1_, the *V*_out_ decreases to the response value dominated by the H_2_S oxidation and CuS formation behaviors collectively. After being exposed with air at *t*_2_, the *V*_out_ rises to a platform within 150 s on account of the oxygen re-adsorption process. The recovery platform is defined as recovery I. This first recovery process is affected by the CuS formation since CuS covering on the CuO surface can reduce the chemically active sites for oxygen molecules’ chemisorption. Next, *V*_out_ cannot restore the baseline value due to the existence of CuS, since the Cu–S bond is too strong to break at a low temperature. Overall, CuS formation clearly hinders the recovery process of the device. After applying a heating pulse (350 °C, 30 s), *V*_out_ quickly rises up to the baseline state. The schematic illustrations related to the sensing process are present in Figure 7b. In this case, the vertical height between the response value and baseline value is marked as *h*, the vertical height between the response value and the recovery I is recorded as *h*_1_, and the ratio of *h*_1_/*h* is marked as *k*%, which represents the proportion of the recovery I in the whole recovery process. The smaller (greater) the ratio is, the stronger the inhibition on the device recovery process by CuS formation is.

**Figure 8:**
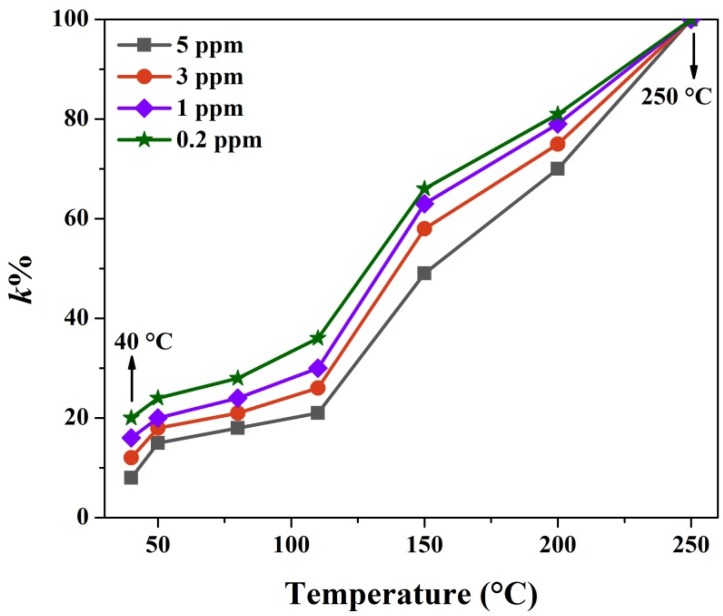
The *k*% values on different operating temperatures at various H_2_S concentrations.

**Figure A2:**
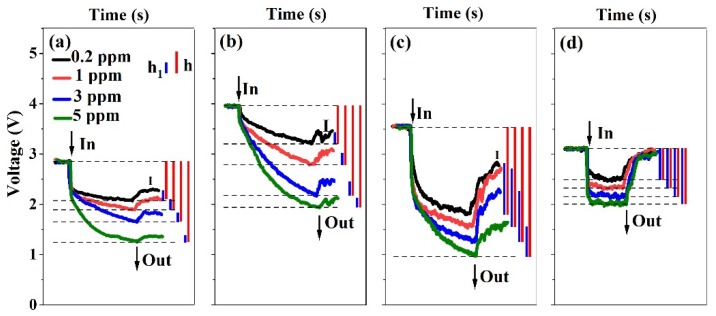
Dynamic performances on time of CuO sensor toward 0.2, 1, 3, and 5 ppm H_2_S gas at (**a**) 40 °C, (**b**) 80 °C, (**c**) 150 °C, and (**d**) 250 °C.

Figure A2 depicts the dynamic curves of the sensor at different H_2_S concentrations and operating temperatures. To exhibit the dependence of *k*% values on working temperatures and gas concentration intuitively, the *k*% versus the two factors was shown in Figure 8. As seen, the *k*% decreases while increasing H_2_S concentrations from 0.2 ppm to 5 ppm. Such a variation indicates that the inhibition on the recovery process by CuS formation enhances with the expanded coverage of H_2_S molecules. In addition, the k% increases as the temperature rises from 40 °C to 250 °C. Therefore, the inhibition of CuS formation on the recovery process becomes weak. Additionally, when the temperature rises to 250 °C, the *k*% is approximately 100% toward different concentrations of H_2_S gas. This indicates that CuS formation has no effect on the sensor recovery because the high temperature facilitates the complete oxidation of CuS to CuO. The generated CuS (according to Equation (2)) was converted to CuO simultaneously (based on Equation (4)). Ultimately, the CuS formation cannot be detected since the two reactions achieve equilibrium at such a high temperature. 

**Figure 9:**
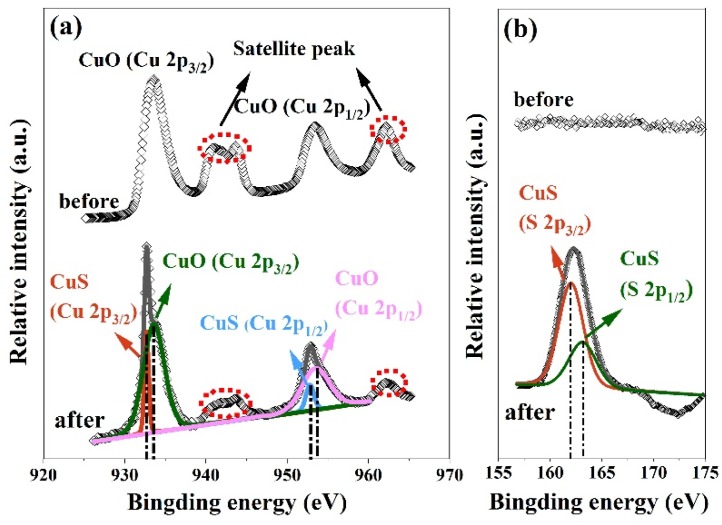
(**a**) Cu 2p spectrum and (**b**) S 2p spectrum of CuO nanoparticles before and after H_2_S sensing.

The XPS measurement was adopted to confirm the CuS formation, as illustrated in Figure 9a. Before exposure to H_2_S gas, the binding energies at 933.6 eV and 953.5 eV are due to Cu 2p_3/2_ and Cu 2p_1/2_ for CuO, and the two satellite peaks at ~943 eV and ~963 eV relate to the paramagnetic chemical state of Cu^2+^ [32,33]. In the case of H_2_S exposure, the two satellite peaks still exist. Meanwhile, the Cu 2p_3/2_ peak has two components at 932.7 eV and 933.6 eV, and the Cu 2p_1/2_ peak is due to two components at 952.8 eV and 953.5 eV, respectively. The positions of 932.7 eV and 952.8 eV are typical peaks for CuS [34,35], and the positions of 933.6 eV and 953.5 eV are corresponding to the peaks of CuO [36,37], which verifies the generation of CuO/CuS composites. Furthermore, Figure 9b explores that the S 2p peaks is absent before H_2_S injection, while the S 2p peak appears with two components at 162.0 eV and 163.1 eV after H_2_S exposure. The above positions are attributed to the S 2p_1/2_ and S 2p_3/2_ states in the metal sulfide bond [36,37], which also confirms the CuS formation. Therefore, the XPS results adequately support that CuO is partly conversed into CuS after H_2_S sensing.

**Figure 10:**
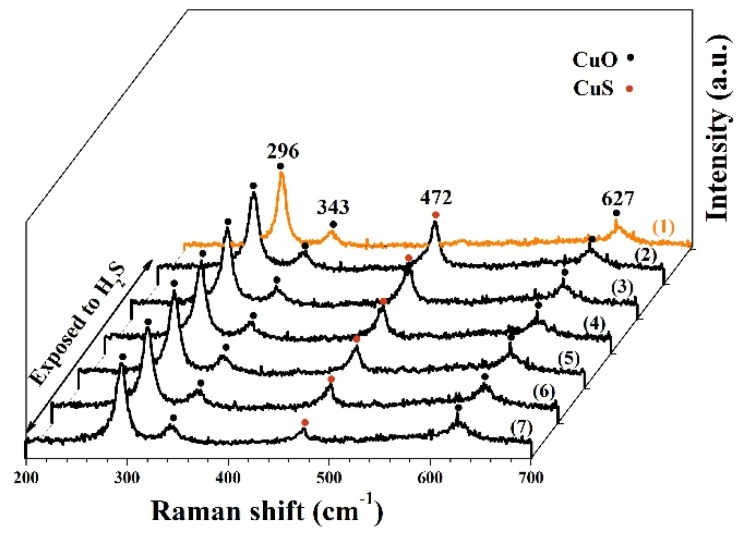
Raman spectra of CuO nanoparticles’ sensor measured by 1 mW laser radiation (1) exposed to air, exposed to H_2_S gas after being irradiated by the laser with different energies (2) 1 mW, (3) 2 mW, (4) 3 mW, (5) 4 mW, (6) 6 mW, and (7) 8 mW.

Raman spectra were exploited to modulate the sensor’s dynamic process with temperature. All the Raman spectra were measured by 1 mW laser radiation, as displayed in Figure 10. Curve (1) is raw Raman data of CuO. There are peaks at 296, 343, and 627 cm^−1^ [38,39]. After exposure to H_2_S gas, there is a new sharp peak in 472 cm^−1^ in curve (2). The new peak agrees well with reported values for Cu–S bonding [40,41]. As previously discussed, a short-time heating pulse facilitates the break of Cu–S bonds and finishes the recovery process. To simulate the heating effect on CuS decomposition, the power of the laser radiation was increased from 1 mW to 8 mW to simulate the electric heating pulse. As we know, the local temperature of the area under laser irradiation could be very high. After applying the “heating pulse,” we reduced the laser energy to 1 mW again and then measured the Raman signals. With the increasing laser power, the intensity of the CuS Raman peak slowly decreases and becomes almost unobservable, as illustrated by curves (2)–(7). The micro Raman results support that high temperature facilitates the Cu–S bonding break and, hence, accelerates the sensor’s recovery process.

**Figure 11:**
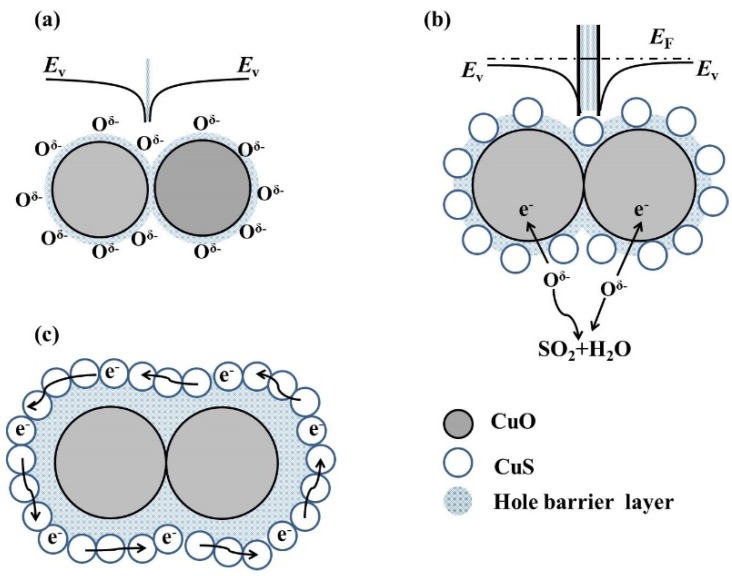
Schematic diagrams of the CuO nanoparticles sensor (**a**) in air and exposed to (**b**) low concentration and (**c**) high concentration of H_2_S gas.

The sensing mechanism of CuO nanoparticles’ sensor to H_2_S gas is described in Figure 11. As presented in Figure 11a, many oxygen molecules in air are chemisorbed on the surfaces of tiny-grain CuO based on depletion theory. These adsorbed oxygen molecules become ionized oxygen species O^δ−^ by grasping free electrons from the conduction band of the CuO nanoparticles. The process of capturing electrons leads to the construction of a Schottky-barrier between the inner grains and the formation of a hole barrier layer in the near-surface region, which is analogous to the inter-granular contacts in high-porosity SnO_2_ [42,43]. As shown in Figure 11b and Equation (1), once the CuO nanoparticles contact each other with a low concentration of H_2_S gas, the chemisorbed O^δ−^ reacts with H_2_S to be transformed into H_2_O and SO_2_. Those electrons previously trapped by oxygen atoms are instantaneously released back into the conduction band of CuO [44], which leads to a decrease in carrier density and an increase in sensor’s resistance. Meanwhile, CuO is partly converted into CuS with lower work function [21,45]. Electrons flow from CuS to CuO. The hole barrier layers on the CuO surface are enhanced relative to that in air, so CuS formation behaviour further amplifies the sensor’s resistance. However, it is reported that there is a significant reduction in electrical resistance after CuO is sulfurized to CuS [18,22] because CuS has a metallic character with a good conductor and low resistance [12,20]. The different results depend on the H_2_S concentration. When the H_2_S concentration further increases, the covering region of CuS on the CuO surface grows gradually, and eventually becomes a continuous CuS covering layer that facilitates the carrier flows directly from the surface itself. This would lead to a significant reduction in the sensor’s resistance. The schematic diagram is illustrated in Figure 10c. In addition, the mechanism analysis can explain the abnormal phenomenon of devices reacting to high concentration of H_2_S gas, as presented in Figure 6.

## 4. Conclusions

CuO nanoparticles were synthesized using a facile one-step hydrothermal method. Gas sensors fabricated by CuO nanoparticles exhibit a sensitive response, good selectivity, and fast recovery to H_2_S gas when applying a heating pulse at the best working temperature of 150 °C. Additionally, even at 40 °C, the sensing performance is still good enough for monitoring the application. The dynamic curves upon H_2_S exposure/release process display the synergistic effect of H_2_S oxidation mechanism and CuS formation mechanism to the total response. The inhibition effect of CuS formation on the device recovery process depends on the working temperature and gas concentration. The chemical conversion of CuO to CuS was observed from the XPS results of CuO nanoparticles during H_2_S sensing. The CuS bond decomposition by a thermal effect was verified by Raman analysis. The results provide a feasible guidance for a deeper understanding of the response mechanism of CuO materials to H_2_S gas. 

## Figures and Tables

**Table 1:** A comparison of H_2_S gas sensing performances based on CuO-based nanostructures.

Materials	Concentration (ppm)	Temperature	Response	Response Time (s)	Recovery Time (s)	Reference
Cu_2_O/CuO Cu_2_O	0.1 0.1	95 °C RT	2.5 1.7	76 100	75 /	[2] [9]
CuO thin film	5	RT	3.5	>100	>4000	[26]
CuO nanoparticles	5	40 °C	4.9 ± 0.43	297.5 ± 9.2	54 ± 7.1	This work
